# H1N1 Influenza Virus-Infected Nasal Mucosal Epithelial Progenitor Cells Promote Dendritic Cell Recruitment and Maturation

**DOI:** 10.3389/fimmu.2022.879575

**Published:** 2022-04-28

**Authors:** Fangyuan Zhu, Zhenxiao Teng, Xuanchen Zhou, Runtong Xu, Xin Bing, Lei Shi, Na Guo, Min Wang, Chengcheng Liu, Ming Xia

**Affiliations:** ^1^Department of Otolaryngology, Shandong Provincial Hospital Affiliated to Shandong First Medical University, Jinan, China; ^2^Department of Otolaryngology, Shandong Provincial Hospital, Cheeloo College of Medicine, Shandong University, Jinan, China; ^3^Department of Pathology, Shandong Provincial Hospital Affiliated to Shandong First Medical University, Jinan, China; ^4^Central Laboratory, Shandong Provincial Hospital Affiliated to Shandong First Medical University, Jinan, China

**Keywords:** influenza A virus, multiomics analysis, immune response, arginine metabolism, nasal mucosal epithelial cells

## Abstract

The barrier function of nasal mucosal epithelial cells plays an irreplaceable role in the spread and expansion of viruses in the body. This study found that influenza A virus H1N1 could induce apoptosis of nasal mucosal epithelial progenitor cells, cause an inflammatory response, and trigger the maturation and recruitment of nasal submucosal dendritic cells (DCs), but the mechanism remained unclear. Therefore, we used RNA sequencing and high-resolution untargeted metabolomics to sequence and perform combined bioinformatic analysis of H1N1 virus-infected nasal mucosal epithelial cells from 6 different patients. The abnormal arginine metabolism signaling pathway caused by H1N1 virus infection was screened out, and arginase inhibitors were used to interfere with the abnormal arginine metabolism and the maturation and recruitment of submucosal DCs caused by the H1N1 virus *in vitro* and *in vivo*. We conclude that H1N1 influenza virus promotes the recruitment and maturation of submucosal DCs by causing abnormal arginine metabolism in nasal mucosal epithelial cells, thereby triggering respiratory mucosal immunity.

## Introduction

Respiratory viral infection is one of the most common diseases in humans, and deterioration of the disease can lead to various complications, which seriously threaten people’s life and health (1). About 90% of upper respiratory tract infections is caused by viruses. In recent years, some new types of highly pathogenic viruses that mainly cause respiratory tract infections have emerged, such as influenza A virus, which is a negative-sense enveloped RNA virus and hemagglutinin (HA) and neuraminidase (NA) are antigenic glycoproteins on the surface of the virus, and the pathogenicity of the virus is directly related (2, 3). Influenza A virus H1N1 is one of the main viruses that cause acute upper respiratory tract infections in humans and animals, with high morbidity and mortality worldwide. The H1N1 virus is a single-stranded, negative-sense, segmented RNA, which can encode 10 proteins, including M1, M2, NS1, and NS2 (4, 5). It has been shown that many respiratory viruses, such as rhinovirus and respiratory syncytial virus (RSV), can lead to loss of epithelial cell barrier function by impairing the integrity of the respiratory epithelium (6–8). Dysfunction of the epithelial cell barrier plays an irreplaceable role in the spread and expansion of the virus in the body.

The epithelium of the nasal mucosa is the first physical barrier between the body and the external environment, and it plays an important role in preventing pathogens, such as bacteria, viruses, and allergens, from invading the body (9). Normally, the nasal mucosal epithelial cells comprise ciliated cells, goblet cells, columnar cells, and basal cells (10). The barrier function of the epithelium depends on the integrity of epithelial cells and the junctional complex between cells. After the influenza virus breaks through the mucosal barrier, it adsorbs and invades the nasal mucosal epithelial cells, and it blocks the signaling pathways in the pathogen recognition system (PRRs) of the nasal mucosal epithelial cells; thus, the immunosuppressive mechanism is disrupted (11). Subsequently, the virus rapidly replicates and spreads in the immune cells, inducing immune activation, which in turn leads to a pro-inflammatory or an acute inflammatory response (12, 13). Therefore, the nasal mucosal barrier is a key barrier to influenza virus uptake and subsequent antigen-specific adaptive immune responses.

Dendritic cells (DCs) play a crucial role in the innate immune response and subsequent adaptive immunity (14). These cells highly express pattern recognition receptors (PRRs), which can produce chemokines to recruit other immune cells, such as monocytes and neutrophils, when infected by viruses, and they stimulate epithelial cells to carry out cytokine cascade amplification, thereby rapidly causing a severe inflammatory response (15). Submucosal DCs, sentinel cells underneath the nasal epithelium, are essential for acquiring and presenting foreign antigens, which are a prelude to the initiation of adaptive immune responses (16). After antigen challenge, DCs enter the maturation process and migrate to lymphoid organs, thereby effectively stimulating T cell activation. Therefore, DC maturation is the first critical step in the subsequent antigen-specific immune response. Studies have shown that CD103 DCs can respond to viral (including influenza virus) stimulation in the lung, carry out antigen capture and presentation, and induce immune system activation (17). However, it remains unclear how DC recruitment and maturation play a role in nasal mucosal immunity.

Nasal mucosal epithelial cells undergo necroptosis after virus infection, release a large number of cytokines, and cause a cytokine storm in the body (18). Mass release of cytokines can stimulate the activation of a variety of immune cells, and these types of immune cells are very important for induction of the host’s innate immune response. Different cytokines (including interferons (IFNs), interleukins (ILs), chemokines, colony-stimulating factors, and tumor necrosis factor (TNF)) exert many different functions, which act on different target cells by binding to specific receptors alone or in combination with other cytokines, thereby inducing a variety of responses (19–21). For example, IL-10 can promote the differentiation of naive CD4 T cells into Th2 cells, IL-12 can enhance CD8 T cells and non-specific cell-mediated immune responses, and TNF-α, which is a pro-inflammatory cytokine, can stimulate DC maturation (22–24). However, it remains unclear how H1N1 virus infection causes nasal mucosal epithelial damage and the downstream immune response.

In many acute severe infections, although a variety of anti-inflammatory drugs and adjuncts are used to modulate the body’s response, clinical intervention is ineffective and symptom improvement is not achieved. Therefore, the purpose of this study is to use a variety of omics combined analysis methods to explore the molecular mechanism of nasal mucosal epithelial cell damage and regulation during H1N1 virus attack, as well as to assess the regulatory effect of H1N1 virus on downstream DC cells through nasal mucosal epithelial cells in order to identify more effective and precise targeted therapies for different stages of the immune cascade.

## Materials and Methods

### Subjects

The patient-related experiments in this study were approved by the Ethics Committee of Shandong Provincial Hospital Affiliated to Shandong First Medical University (No. 2020-354), and they were conducted in accordance with the relevant guidelines and regulations. *In vitro* cultured human nasal epithelial stem/progenitor cells (hNESPCs) were obtained from 6 patients with a deviated nasal septum who underwent septoplasty at Shandong Provincial Hospital, China. All subjects did not have any symptoms of upper respiratory tract infection. **Table 1** summarizes the characteristics of patients included in this study.

**Table 1:** The characteristics of donating patients.

Number	Age	Gender	Smoking history	Diagnose	Specimen
1	34	female	non-smokers	septal deviation	inferior turbinate
2	52	Male	non-smokers	septal deviation	inferior turbinate
3	29	Male	non-smokers	septal deviation	inferior turbinate
4	37	Male	non-smokers	septal deviation	inferior turbinate
5	45	Male	non-smokers	septal deviation	inferior turbinate
6	26	female	non-smokers	septal deviation	inferior turbinate

The animal experiments performed in this study were also approved by the Ethics Committee of Shandong Provincial Hospital Affiliated to Shandong First Medical University (No. 2020-443). All animal experiments followed the laws and regulations of animal protection. Animals were kept in a controlled sterile environment with constant temperature and humidity, and sufficient food and water were provided.

### Reagents

Rabbit polyclonal anti-Bax (Cat. No.A19684), anti-Caspase 3(Cat. No. A11319) and anti-Actin (Cat. No. AC026) antibodies were purchased from Abclonal (Wuhan, China), and rabbit polyclonal anti-Bcl2 antibody (Cat. No. 12789-1-AP) was obtained from Proteintech (Wuhan, China). Anti-CD11c antibody (Cat.No.ab52632) was obtained from Abcam (Cambridge, United Kingdom). Arginase inhibitor 1 (Cat.No.1345808-25-4) was obtained from MedChemExpress (New Jersey, United States).

### Cell Culture

Primary human nasal epithelial cells **(**hNEPCs) were maintained in Dulbecco’s Modified Eagle Medium (DMEM)/Nutrient Mixture F-12 (Cat.No.11320033, Gibco), which contained human epithelial growth factor (Cat.No.100-15-500, Peprotech), insulin (Cat.No.I5500-50MG, Sigma), cholera toxin (Cat.No.C9903-0.5MG, Sigma), hydrocortisone (Cat.No.H0888-1G, Sigma), 3,3’,5-triiodo-l-thyronine (Cat.No.T6397-100MG, Sigma), and N-2 supplement (Cat.No.17502001,Gibco-Invitrogen). Further, a 1% Antibiotic-Antimycotic solution (Cat. No.MIR 5970, Mirusbio) was added. hNEPCs were cultured at 37°C in a 5% CO2 cell incubator.

The NIH/3T3 cell line purchased from American Type Culture Collection (ATCC, Manassas, VA, USA) was cultured in DMEM (Cat.No.C11995500BT, Gibco) containing 10% fetal bovine serum (FBS) (Cat. No. 10099-141, Gibco-Invitrogen) and 1% penicillin-streptomycin (Cat. No.15140-122, Gibco). When the cell density reached about 80%, mitomycin C (Cat.No.M4287-2MG, Sigma) was added, treated in a 37°C cell incubator for 2 h, washed three times with PBS, and then co-cultured with the isolated primary cells.

Madin-Darby canine kidney (MDCK) cells were cultured in the following manner: 5% fetal bovine serum (FBS), 100 IU/mL penicillin, 100 mg/ml streptomycin, and 2 mM glutamine were added to the DMEM. Further, the cells were cultured at 5% CO2 and 37°C.

### hNEPC Isolation and Culture, and Human Nasal Epithelial Cell (hNEC) Differentiation

The inferior turbinate mucosal tissue collected during endoscopic surgery was placed in Hanks’ salt-balanced solution containing 100 IU/ml of antibiotics and antifungals within 1 hour. The tissue was cut into small pieces with sterile scissors on an ultra-clean bench, and 1 mg/ml type IV collagenase (Gibco) was added for digestion at 37°C and 5%CO2 concentration for 2 h. Digestion in the medium was terminated and primary cells were isolated after repeated purging, centrifugation, and filtration through a 100 um filter. The isolated primary epithelial progenitor cells were cultured in a specially prepared medium containing adherent trophoblast cells (NIH3T3 cells).

Expanded hNESPCs were transferred to 12-well 0.4 μm Transwell inserts (Corning, Corning, NY, USA) containing 1000 μl of B-ALITM growth medium (Lonza, Walkersville, MD, USA), which were added to the basal chamber. After three days, the growth medium was carefully discarded with a pipette and 1000 µL of B-ALITM differentiation medium was added to the basal chamber to establish air-liquid surface culture conditions. Cells were cultured continuously for 5 weeks under air-liquid surface conditions, with medium changes every 2 days. After 5 weeks of cell differentiation, H1N1 influenza virus infection experiments were performed.

### Influenza Virus A H1N1 Infected Mice

Forty C57BL/6 male mice aged 4-6 weeks were randomly divided into 4 groups, 10 mice in each group, 5 mice in each cage, and were marked separately. After the mice were anesthetized by ether inhalation, they were infected with 10^6^ TCID50 virus solution by nasal instillation, 50ul per mouse, and the control mice received the same amount of saline intranasally. It was raised normally after four hours. Mice were sacrificed on day 5 after viral infection and tissues were taken for further mechanistic studies.

### Influenza Virus A H1N1 Infection of hNEPCs (or hNECs)

The H1N1 virus strain is A/California/7/2009. Viral allantoic fluid was collected after the proliferation of chicken embryos without specific pathogen free (SPF) with an TCID50 of 107. -80°C Storage for backup. Before infection, hNECs were washed three times with PBS buffer and incubated with H1N1 influenza virus at 37°C for 2 h. After 2 h of incubation, the viral inoculum was removed, and the cells were incubated at 37°C for 8 h, 16 h, and 24 h, and the infected hNECs were harvested for subsequent experiments.

### Mice Were Injected With Arginase Inhibitor 1

Arginase inhibitor 1 is freshly formulated in 0.9% saline prior to dosing, at 10 mg/mL for i.v. dosing at a dose volume of 1 mL/kg animal body weight,mice are fasted overnight prior to dosing, with water given ad libitum. Arginase inhibitor 1 is administered i.v. through a preimplanted cannula. Food is reintroduced to animals 4 h following dosing.

### Plaque Assay

At each collection time point of infected hNECs, 150 µL of 1x PBS was added and cells were incubated in a 37°C incubator for 10 min to recover progeny virus in the apical wash. Viral quantitative plaque assays were performed when MDCK (ATCC, Manassas, VA, USA) cells were grown to 85-95% confluency. Further, 100 µl of serial dilutions (10 ^- 1^ to 10 ^- 13^) of apical wash of infected hNECs were added to MDCK cells and incubated at 37°C for 1 hour. After incubation, cells were fixed with 4% formaldehyde for 30 min, stained with crystal violet, and the plaques were counted after washing off the unstained dye using the following formula:


Number of plaques×dilution factor= numberofPFUper100μL.

### RNAseq Analysis

Total RNA was isolated from hNECs using the Trizol Reagent (Invitrogen Life Technologies), after which the concentration, quality, and integrity were determined using a NanoDrop spectrophotometer (Thermo Scientific). The mRNA was purified from total RNA using poly-t oligosaccharide linked magnetic beads. The first-strand cDNA was synthesized using random oligonucleotides and superscript II. The second-strand cDNA was then synthesized using DNA polymerase I and RNase H. The remaining overhang was converted to a blunt end by exonuclease/polymerase activity and the enzyme was removed. In order to select cDNA fragments with a preferred length of 400-500 BP, the library fragments were purified using the Ampure XP system (Beckman Kurt, Beverly, California, USA). In 15 cycles of PCR reaction, DNA fragments with connecting junction molecules at both ends were selectively enriched using Illumina PCR primer mixture. The product was purified (ampoule XP system) and quantified by Agilent high-sensitivity DNA analysis on the biological analyzer 2100 system (Agilent). Multiple test corrections were performed using the methods of Benjamini and Hochberg, and p-values (False Discovery Rate; FDR) less than 0.05 were considered significant.The original contributions presented in the study are publicly available. This data can be found here: https://www.ncbi.nlm.nih.gov/sra/PRJNA813174.SRA data is PRJNA813174.

### High-Resolution Untargeted Metabolomics Analysis

High-resolution non-targeted metabolomic analysis was performed on hNECs treated with H1N1 virus infection for different times. Ultrahigh performance liquid chromatography tandem time-of-flight mass spectrometry (UHPLC-Q-TOF MS) was used to detect the metabolites in the samples. The metabolites in the biological samples were identified by matching the retention time, molecular weight (molecular weight error < 10 ppm), secondary fragmentation spectrum, collision energy, and other information of the metabolites in the local database, the appraisal results were checked strictly and were confirmed manually.

### Co-Culture of hNECs and DCs

hNECs were seeded into the upper compartment of the Transwell chamber, and they formed a differentiated and mature multicellular epithelial layer after 35 days of continuous culture in the upper chamber. When hNECs were infected with H1N1 virus for 24 hours, DC cells(derived from mouse bone marrow) were placed in the lower chamber of the chamber. Since the transmembrane barrier prevents direct contact between DCs and hNECs, this model allows the study of activation of DCs by soluble factors released by virus-infected epithelial cells. The cell co-culture model is shown in **Figure 3**.

### Flow Cytometry

To detect the level of apoptosis, H1N1 virus-infected hNECs were collected, and the Annexin V-FITC/PI Kit (Cat. No. 556547, BD) was used. Apoptosis levels of hNECs were assayed according to the manufacturer’s instructions.DCs co-cultured with hNECs, DCs or nasal lavage, bronchoalveolar lavage, triturated filtered lymphoid cells from mice were collected, digested, centrifuged, and resuspended in PBS. After the cells were fixed and washed, PE-conjugated anti-CD11c antibody (Cat. No.566730, BD), APC-conjugated anti-CD86 antibody (Cat. No. 305412, Biolegend), FITC-conjugated anti-CD80 antibody (Cat. No. 375406 Biolegend), PE-CF594-conjugated anti-MHCII antibody (Cat. No. 564807, BD), FITC-conjugated IFN-γantibody (Cat. No. 552887, BD), or PE-conjugated IL-4 antibody (Cat. No. 559333, BD) was added to the prepared cells, and they were incubated at room temperature for 30 min in the dark. Detection was performed with a BD FACS flow cytometer and analysis was performed with QuestPro software version 5.1 (BD Bioscience Company, Franklin Lakes, NJ, USA).

### Enzyme-Linked Immunosorbent Assay (ELISA)

ELISA kits were obtained from Beyotime for measuring human IL-6, TNF-α, macrophage chemotactic protein-1 (MCP-1), macrophage inflammatory protein (MIP)-1α, and IFN-α (Cat. No. PI330, Cat. No. PT518, Cat. No. PC130, Cat. No. PC145, and Cat. No. PI505). ELISA kits for detection of human IL-17A, transforming growth factor-beta (TGF-β), and CXCL10 were purchased from Abcam (Cat. No. ab216167, Cat. No. ab100647, and Cat. No. ab83700). ELISA kit for detection of human MIG was purchased from GeneTex (Cat. No. GTX37016). ELISA kits for measuring mouse IgA were purchased from Abcam (Cat. No. ab 157717). The hNEC supernatants were collected at different time points of 8h, 24h, 48h, and 72h after virus infection, and the experimental operation was carried out according to the manufacturer’s instructions for each ELISA kit. Finally, the absorbance of the samples was read at 450 nm using a microplate reader (Multiskan-FC, Thermo Scientific).

### Immunofluorescence

After the co-culture of hNECs and DCs, the bottom of the upper chamber of the Transwell was taken with the lower side facing up, and the cells were washed three times with PBS. Then, it was fixed in 4% paraformaldehyde for 20 min. Cells were permeabilized with 2% TritonX-100 for 10 min, blocked with 2% bovine serum albumin (BSA), and CD11c antibody was added overnight at 4°C. The following day, the cells were incubated with the corresponding Alexa Fluor 594-conjugated secondary antibody for 1 h at room temperature, followed by nucleus staining with 4’,6-diamidino-2-phenylindole (DAPI). Finally, observation was performed with a confocal microscope (LSM 700, Zeiss).

### Cell Viability Assay

The methylthiazolyl tetrazolium (MTT) method was used to measure cell viability. The cell density of hNEPCs in a 96-well plate was 1x 10^4^ cells/well. At 24h and 48h after hNEPCs were infected with H1N1 virus, 20 µl of MTT reagent (5 mg/ml) was added to each well and incubated at 37°C for 4h. Afterwards, the supernatant was gently aspirated from the wells, avoiding touching the bottom of the 96-well plate, and 100 µl of dimethyl sulfoxide (DMSO) solution was added to each well. The 96-well plate was shaken for 10 min at room temperature, and then its absorbance was measured at 570 nm with a microplate reader (Bio-Rad Laboratories, Hercules, California, USA).

### Western Blot

After hNEPCs were infected with H1N1 virus, the cells were collected at different time points of 0h, 2h, 4h, 8h, 16h, 24h, 48h, and 72h, and they were incubated with RIPA lysis buffer containing protease inhibitor for 20 min on ice for cell lysis. Subsequently, the supernatant was collected by centrifugation at 4°C, and the protein concentration was measured using the Bicinchoninic acid (BCA) kit. After denaturing the obtained cell lysate supernatant, proteins were separated by 12% sodium dodecyl sulfate-polyacrylamide gel electrophoresis (SDS-PAGE). The separated proteins were then electroblotted onto polyvinylidene fluoride (PVDF) membranes using a blotting apparatus (Bio-Rad Laboratories). After blocking with 5% skim milk, the membrane was incubated with the corresponding primary antibody at 4°C overnight, and then it was labeled with a suitable secondary antibody for 1 h at room temperature. β-actin was used as an internal reference. Finally, protein signals were detected using the enhanced chemiluminescence (ECL) system (Cell Signaling Technology).

### Real-Time Quantitative PCR

Trizol (Invitrogen) was used to isolate total RNA from hNECs following H1N1 virus infection, and the RNA quality was determined by absorbance at 260 nm and 280 nm. cDNA synthesis was performed using RevertAidTM M-MuLV RT (Fermentas, Hanover, MD, USA) according to the guidelines and stored at -80°C. Quantitative RT-PCR (qRT-PCR) was performed according to the instructions of the Platinum SYBR Green (Invitrogen) kit using the cDNA obtained by reverse transcription as a template. The cycle length of the qRT-PCR reaction was set to 40 cycles, the annealing temperature range was set between 52°C and 66°C, and β-actin was used as a housekeeping gene. The final obtained results were calculated and analyzed using the 2-ΔΔCt method, and all experiments were performed in triplicate. The specific primer sequences are shown in **Table 2**.

**Table 2:** Sequences of siRNA and qPCR primers used in this study.

Gene	Forward primer	Reverse primer
IL-6	5´-TTCTCTGGGAAATCGTGGAAA-3´	5´-TGCAAGTGCATCATCGTTGT-3´
IL-17A	5´-ATCGCTCTGTGATCTGCGAG-3´	5´-CGGACTGTGATGGTCAACCT-3´
TNF-α	5'- TATCCTGGGGGACCCAATGT-3'	5'-AGCTTCTTCCCACCCACAAG-3'
TGF-β	5´-CTCCCGTGGCTTCTAGTGC-3´	5´-GCCTTAGTTTGGACAGGATCTG-3´
CXCL10	5′-AGCAAGGAAAGGTCTAAAAGATCTCC-3′	5′-GGCTTGACATATACTCCATGTAGGG-3′
MCP-1	5’-AGCAGCAAGTGTCCCAAAGA-3’	5’-GGTGGTCCATGGAATCCTGA-3’
MIG	5'-TTTCGAGCTCAAGTCCACACCAACATCTG-3’	5′-TCGCAAGCTTAGAAfGGAGTTCCAAGTCAC-3′
IFN-α	5′- TCCCAGAGGAGGAGTTTG-3′	5′-TTTATGTCGGGAACACGG-3′
TGF- β	5′-CTCCCGTGGCTTCTAGTGC-3′	5′-GCCTTAGTTTGGACAGGATCTG-3′
IL-1α	5′-AATGACGCCCTCAATCAAAGPCG-3′	5′-TGGGTATCTCAGGCATCTC-3′
IL-1β	5′-CAGAAGTACCTGAGCTCGCC-3′	5′-AGATTCGTAGCTGGATGCCG-3′
IL-15	5′-CTTGCCATAGCCAGCTCTTCTTC-3’	5′-CGTGTCTAAGCAGCAGAGTGATG -3’
IL-18	5′-GCACAAGGCACAACAGGCTGC-3’	5′-CAGGTCCTGGAAGGAGCA-3’
IL-10	5′-ATAAAAGGGGGACACCGGGC-3’	5′- CTCATAACCCATGGCTTGGC-3’
CCL8	5′-TGGAGAGCTACACAAGAATCACC-3’	5′-TGGTCCAGATGCTTCATGGAA-3’
CCL22	5′-GGAGGCAAAGAGTAGGGTGTAA-3’	5′-AAATCAGCCAGAAAGGCATAGA-3’
CXCL17	5′-TGCTGCCACTAATGCTGATGT-3’	5′-CTCAGGAACCAATCTTTGCACT-3’
IFN-β	5′-GCACAACAGGTAGTAGGCGA-3′	5′-TGGAAAGAGCTGTCGTGGAG -3
IFN-γ	5′-CTTGGTGGCTGAGTTGGG-3′	5′-ATCGCTGAAGTATGTAATGTAG-3′
MIP-1α	5′-TGCCCTTGCTGTCCTCCTCTG-3′	5′-GGCTGCTCGTCTCAAAGTAGTCAG-3′
Actin	5′- CTCCATCCTGGCCTCGCTGT-3′	5′- GCTGTCACCTTCACCGTTCC-3′

### Statistical Analysis

All data are expressed as the mean ± SD of 3 independently performed experiments. Data analysis was performed using GraphPad Prism Software 9.0 (GraphPad Software, La Jolla, CA, USA). Two-tailed Student’s t-test (normally distributed) or Mann-Whitney U test was used to analyze the significance of the two groups of data. Two-way ANOVA (or mixed model) was used for grouped data analysis. The result was considered significant when the *p* value was less than 0.05. Each data point in the data used for the analysis is expressed as the mean (standard deviation).

## Results

### Effects of H1N1 Virus on hNEC Viability and Plaque Assay

**Figure 1:**
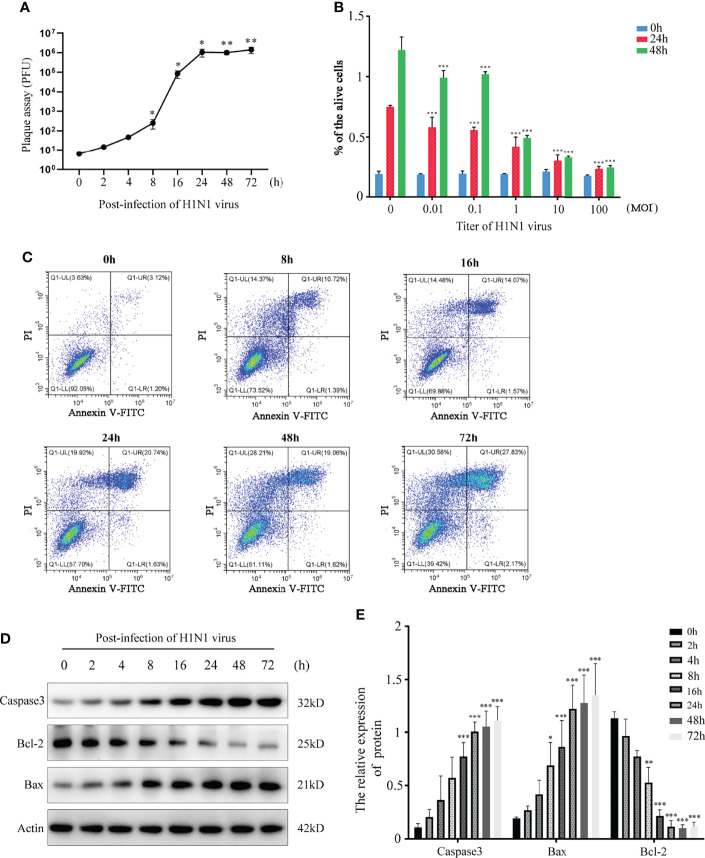
H1N1 influenza virus affects the activity of hNEPCs and promotes apoptosis. **(A)** Viral plaque formation was used to detect the amplification of progeny viruses of H1N1 virus in hNEPCs. **(B)** After 24h and 48h infection with different titers of H1N1 virus, the cell viability was detected by MTT. **(C)** Flow cytometry was used to detect the changes of apoptosis levels after cells were infected with 0.1MOI titer virus for 0-72 hours. **(D)** Detection of apoptosis-related protein expression levels after cells were infected with 0.1MOI titer virus for 0-72 hours.Virus stimulation was increased at different time points, cells were collected at the same time point, and the 0-hour group was the control group. **(E)** The bar graphs show quantification of the apoptosis-related protein expression levels, with each value represents the mean ± SD of three independent experiments. Statistical significance is shown using the Student’s t test analysis, *P < 0.05; **P < 0.01; ***P < 0.001.

Nasal mucosal epithelial cells will induce various emergency responses when attacked by viruses, such as abnormal cell activity, apoptosis, and activation of inflammatory storm. Therefore, we examined the ability of H1N1 virus to expand in nasal mucosal epithelial cells and its effect on the activity of nasal mucosal epithelial cells. We infected human nasal mucosal epithelial cells cultured *in vitro* with H1N1 virus, and detected the formation of plaques in the cells at different times after infection. The results showed that the H1N1 virus could normally expand in the nasal mucosal epithelial cells, and it was in the logarithmic phase of proliferation until 24 hours before infection (**Figure 1A**). Subsequently, we detected the activity and cell proliferation of nasal mucosal epithelial cells after H1N1 virus infection. The results showed that with an increase in the H1N1 virus titer, the viability and proliferation of nasal mucosal epithelial cells gradually decreased, and the cell viability decreased sharply at titers 0.1–1.0 (MOI) (**Figure 1B**). Therefore, the 0.1MOI viral titer was used for subsequent experiments. Studies have shown that viral infection can affect the survival of cells or organisms by increasing the level of apoptosis. Therefore, we also tested the effect of H1N1 virus infection on the apoptosis level of nasal mucosal epithelial cells. Nasal mucosal epithelial cells was stimulated with H1N1 virus at different time periods, but cells were collected at the same time, and took H1N1 virus stimulation for 0 hour as the control group of all groups. The results showed that H1N1 virus infection could induce apoptosis of nasal mucosal epithelial cells, and with the prolongation of infection time, the level of apoptosis tended to increase sharply (**Figures 1C–E**).

### H1N1-Infection Promotes the Secretion of Inflammatory Factors and Chemokines in hNECs

**Figure 2:**
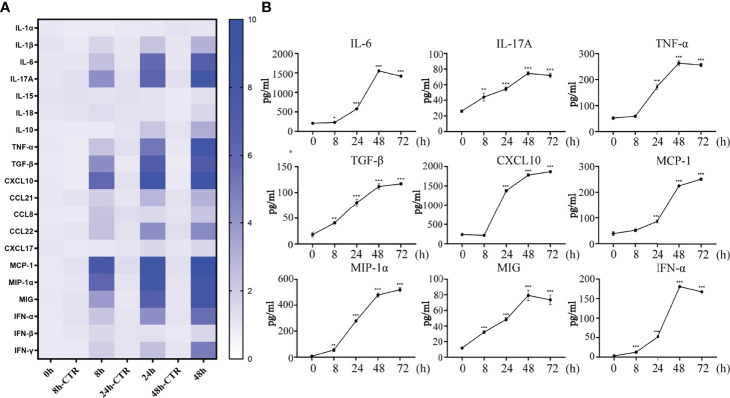
H1N1 virus promotes secretion of cytokines, chemokines and interferons in hNEC cells. **(A)** hNECs were infected with 0.1MOI titer virus for 8 hours, 24 hours, and 48 hours later, the cellular mRNA was extracted, and the expressions of different cytokines, chemokines and interferons were detected at the mRNA level. The histogram of the gradient on the right shows the different folds of change (0~10). **(B)** Cytokines, chemokines and interferons with obvious changes were screened out, and their secretion levels in the culture supernatant were detected by ELISA. Statistical significance is shown using the Student’s t test analysis, *P < 0.05; **P < 0.01; ***P < 0.001.

Cytokines/chemokines (mainly containing interferon) are released after viral stimulation. Cytokinine/chemokine-activated macrophages and virus-infected DCs lead to a broader immune response and the onset of a cytokine storm. In this process, the nasal mucosal epithelial cells bear the brunt. We detected the expression changes in virus-related cytokines, inflammatory factors, chemokines, and interferons in the nasal mucosal epithelial cells after H1N1 virus infection at 8h, 24h, and 48h. The results showed that after the nasal mucosal epithelial cells were attacked by the virus, the expression of chemokines changed most obviously. The β subfamily represents MCP-1, MIP-1α, MIG (CXCL9), and CXCL10, whose expression levels were increased significantly (**Figure 2A**). In addition, the expression levels of the interleukin family members IL-6 and IL-17A, TGF-β, and TNF-α were also significantly increased. Among the members of the interferon family, the expression levels of IFN-α and IFN-γ were increased, but the expression level of IFN-β was not increased (**Figure 2A**). Subsequently, we detected that in the supernatant of the cultured nasal mucosal epithelial cells, the secretion of the significantly increased cytokines, chemokines, and interferons was also significantly increased (**Figure 2B**).

### H1N1-Infected hNECs Promote DC Maturation, Recruitment, and Migration

**Figure 3:**
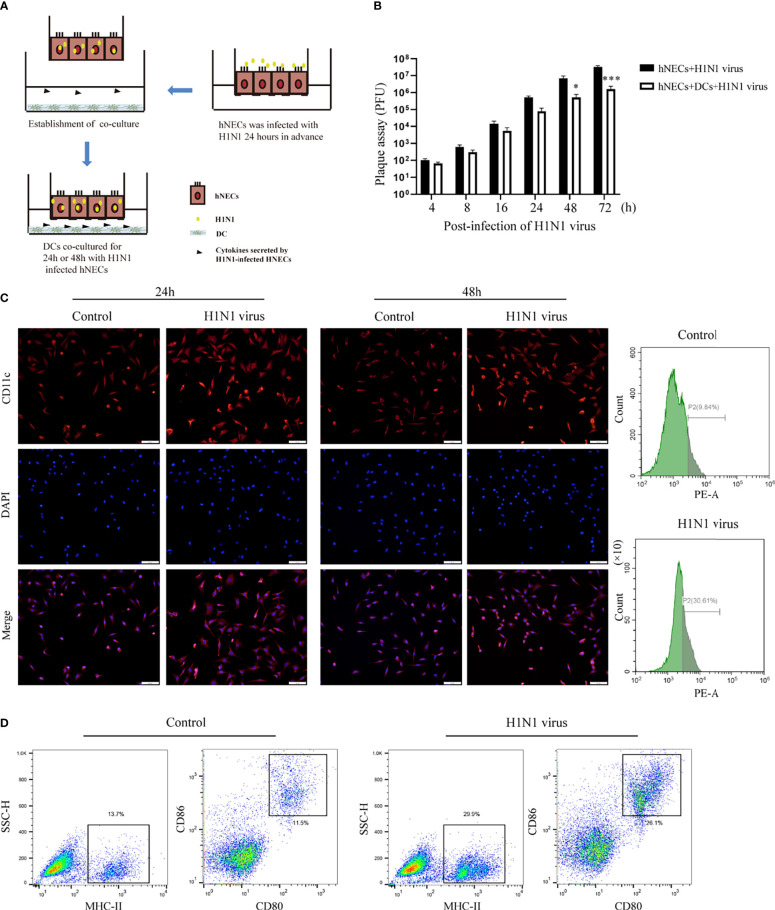
Co-culture of hNEC cells after H1N1 virus infection with DCs induces DCs maturation and recruitment. **(A)** Schematic diagram of the co-culture of hNEC cells after H1N1 virus infection and DCs cells. **(B)** Plaque formation assay was used to detect the expansion of H1N1 virus in hNEC cells alone and hNEC cells co-cultured with DCs cells. **(C)** Immunofluorescence images showed the recruitment of DCs after H1N1 virus-infected and uninfected hNEC cells were co-cultured with DCs for 24h and 48h. The red fluorescence represents CD11c-specific staining, and the blue fluorescence represents nuclear staining (DAPI), bar=50um. The peak graph on the right is the detection of CD11c red fluorescence intensity after 24h co-culture of H1N1 virus-infected and uninfected hNEC cells and DCs cells by flow cytometry. **(D)** Flow cytometry was used to detect the proportion of CD80+CD86+ and MHCII+ cells after co-culture of H1N1 virus-infected and uninfected hNEC cells with DCs cells for 24 hours. Statistical significance is shown using the Student’s t test analysis, *P < 0.05; ***P < 0.001.

Underneath the nasal mucosal epithelial cells, DCs function as immune sentinels by monitoring pathogenic microorganisms, viruses, and allergens. Studies have demonstrated that DCs play an important role in responding to viruses (including influenza virus), allergens, and apoptotic cells. To explore the role of DCs in the defense of nasal mucosal epithelial cells against viral invasion, we co-cultured H1N1 virus-infected nasal mucosal epithelial cells with DC cells (**Figure 3A**). It was found that the presence of DCs significantly reduced H1N1 viral plaque formation in the nasal mucosal epithelial cells after 48 h of co-culture (**Figure 3B**). Subsequently, we detected the migration of DCs after 24h and 48h of co-culture. The results showed that the nasal mucosa epithelial cells infected with H1N1 virus could significantly promote the migration of DCs to the epithelial cells. However, the effect of co-culture for 24h and 48h on DC migration was not significantly different (**Figure 3C**). In addition, we also found that the nasal mucosal epithelial cells infected with H1N1 virus could also promote the expression of CD80, CD86, and MHCII in DCs (**Figure 3D**). This suggests that H1N1 virus infection can promote the maturation of nasal submucosal DCs.

### RNA Sequencing Analysis of H1N1-Infected hNECs

**Figure 4:**
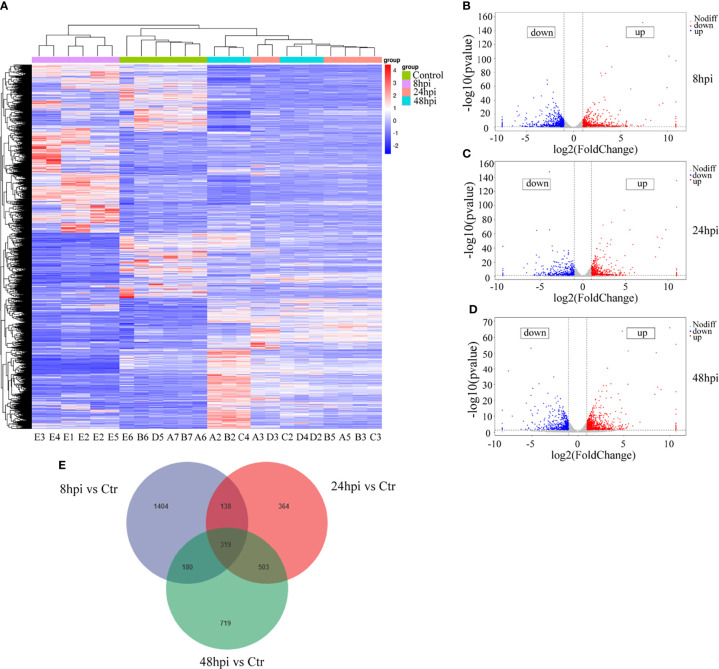
RNA-seq sequencing analysis of H1N1-infected hNEC cells. After 8h, 24h, and 48h of H1N1 virus infection of hNEC cells, the cells were collected for transcriptome sequencing (RNA-seq). **(A)** Heat map showing all differential gene expression profiles of H1N1 virus-infected hNEC cells at 8h, 24h, and 48h compared to control. Each column represents a hNEPCs sample from a different person. **(B)** Volcano plot showing all up- and down-regulated differential genes in hNEC cells infected with H1N1 virus at 8h compared to control. **(C)** Volcano plot showing all up- and down-regulated differential genes in hNEC cells infected with H1N1 virus at 24h compared to control. **(D)** Volcano plot showing all up- and down-regulated differential genes in hNEC cells infected with H1N1 virus at 48h compared to control. Each dot represents a gene, red dots represent up-regulated, blue dots represent down-regulated and grey dots represent no difference. **(E)** Venn diagrams showed that H1N1 virus infected hNEC cells for 8h, 24h, and 48h compared with the control group, and there were 319 overlaps in all differential genes.

In order to identify the reasons for the induction of downstream immune responses and the recruitment and maturation of DCs in the nasal mucosal epithelial cells after H1N1 virus infection, we performed RNA sequencing analysis on nasal mucosal epithelial cells at 8h, 24h, and 48h after virus infection. The sequencing results showed that compared with the control group without virus stimulation, a large number of differentially expressed genes were significantly increased or decreased in the nasal mucosal epithelial cells at 8h, 24h, and 48h after virus infection, and the differential genes that appeared at 24h and 48h of virus infection were closer (**Figure 4A**). We analyzed the differentially expressed genes at 8h, 24h, and 48h. The results showed that the highest number of differentially expressed genes appeared at 8 h of virus infection, of which 1065 genes were up-regulated and 976 genes were down-regulated (a total of 2061 differential genes); the least number of differentially expressed genes were found at 24h of infection, among which 712 genes showed increased expression and 612 genes showed decreased expression (1324 differentially expressed genes in total); at 48h of infection, the median number of differentially expressed genes were found (1721 in total, 1032 up-regulated, and 689 down-regulated) (**Figures 4B–D**). Although the differentially expressed genes differed greatly at different times of infection, there were still 319 overlapping differentially expressed genes (**Figure 4E**).

**Figure 5:**
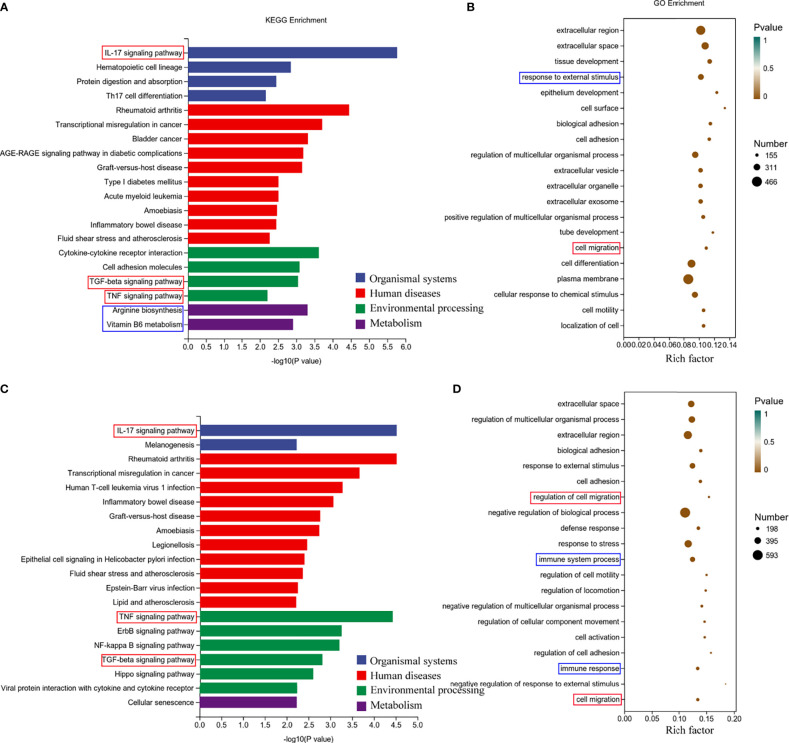
Enrichment analysis of RNA-seq results of H1N1 virus-infected hNEC cells. **(A, B)** Kyoto Encyclopedia of Genes and Genomes (KEGG) pathway **(A)** and Genetic ntology (GO) **(B)** enrichment analysis of H1N1 virus-infected hNEC cells at 24h. **(C, D)** KEGG pathway **(C)** and GO **(D)** enrichment analysis of H1N1 virus-infected hNEC cells at 48h. The color of the bar indicates -log10 (p-value).

Next, we performed Gene Ontology (GO) and Kyoto Encyclopedia of Genes and Genomes (KEGG) analyses of the screened differentially expressed genes. We selected the differential genes at 24h and 48h of virus infection whose distribution was relatively close, and carried out gene enrichment and functional enrichment analysis. Among the gene enrichment analysis results, we were concerned that, in addition to the clustering of differential genes related to cellular components, there were a large number of differential genes related to cell migration and immune response (**Figures 5B, D**). The signaling pathway enrichment analysis showed that the IL-17 signaling pathway was the most significantly changed cytokine-related signaling pathway in the nasal epithelium after 24h and 48h of virus infection. In addition, the TNF and TGF-β signaling pathways also underwent significant changes (**Figures 5A, C**). In addition to this, we noticed that metabolism-related signaling pathways (Arginine biosynthesis and Vitamin B6 metabolism) were also altered (**Figure 5A**). This suggests that H1N1 virus infection may also change the metabolic pattern of nasal mucosal epithelial cells.

### Metabolomic Analysis of H1N1-Infected hNECs

**Figure 6:**
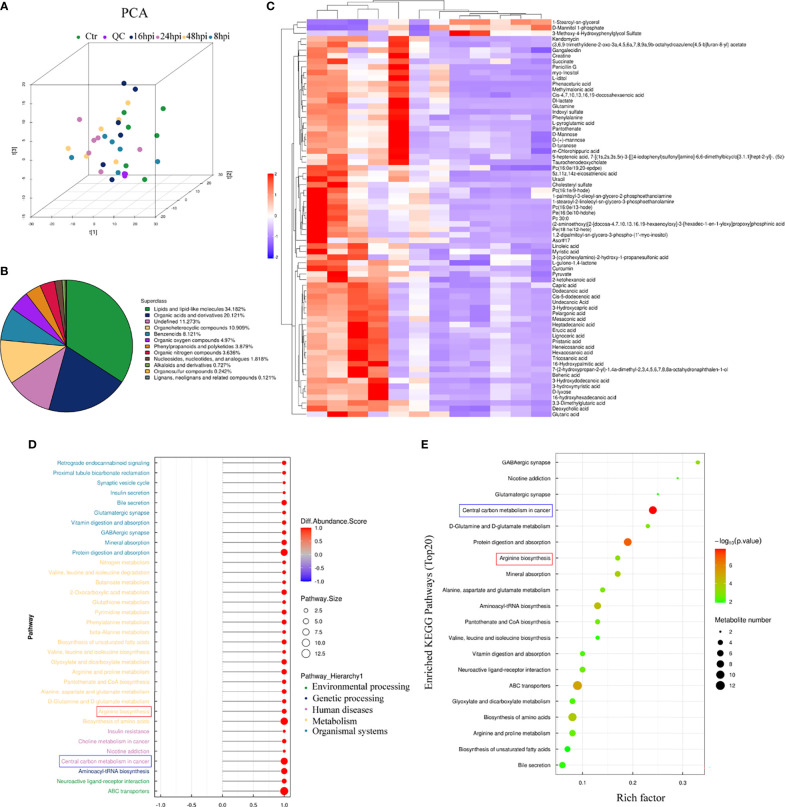
Metabolomic analysis of H1N1-infected hNEC cells. After 8h, 24h, and 48h of H1N1 virus infection of hNEC cells, the cells were collected for High-Resolution Untargeted Metabolomics. **(A)** Population-Sample Principal Component Analysis (PCA) Plot for High-Resolution Untargeted Metabolomics [3D Model]. The virus-infected group at each time point had 6 cell samples from different people. **(B)** All identified metabolites were classified according to their chemical classification information and their proportions were counted. **(C)** Heat map showing all differential gene expression profiles of H1N1 virus-infected hNEC cells at 24h compared to control. Each column represents a hNEPCs sample from a different person. **(D, E)** KEGG pathway **(D)** and GO **(E)** enrichment analysis of H1N1 virus-infected hNEC cells at 24h. The color of the bar indicates -log10 (p-value).

To explore whether H1N1 virus infection affects the metabolic level changes in nasal mucosal epithelial cells, we performed high-resolution non-targeted metabolomic analysis of nasal mucosal epithelial cells after virus infection. Principal component analysis (PCA) analysis was performed on all samples before metabolomic analysis (**Figure 6A**), and a total of 825 metabolites were identified using positive and negative ion modes, and statistical studies were performed according to the chemical classification of metabolites (**Figure 6B**). We screened a total of 72 differentially expressed metabolites for cluster analysis (**Figure 6C**). Subsequently, we carried out the enrichment analysis of the functions and metabolic pathways of the differential metabolites. The results showed that the differential metabolic pathways were mainly divided into environmental processing-related, genetic processing-related, human diseases-related, metabolism-related, and organic systems-related pathways (**Figure 6D**). Among them, arginine biosynthesis was consistent with the previous RNA-sequencing results (**Figure 5A**, **6D, E**). In addition, there was obvious differences in the central carbon metabolism in the cancer metabolic signaling pathway, which also attracted our attention (**Figures 6D, E**). The above results suggest that H1N1-induced changes in the metabolic level of nasal mucosa may be closely related to the secretion of cellular inflammatory factors and regulation of downstream immune cells.

### Arginase Inhibitor 1 Can Regulate the Recruitment of DCs Induced by H1N1 Virus-Infected hNECs

**Figure 7:**
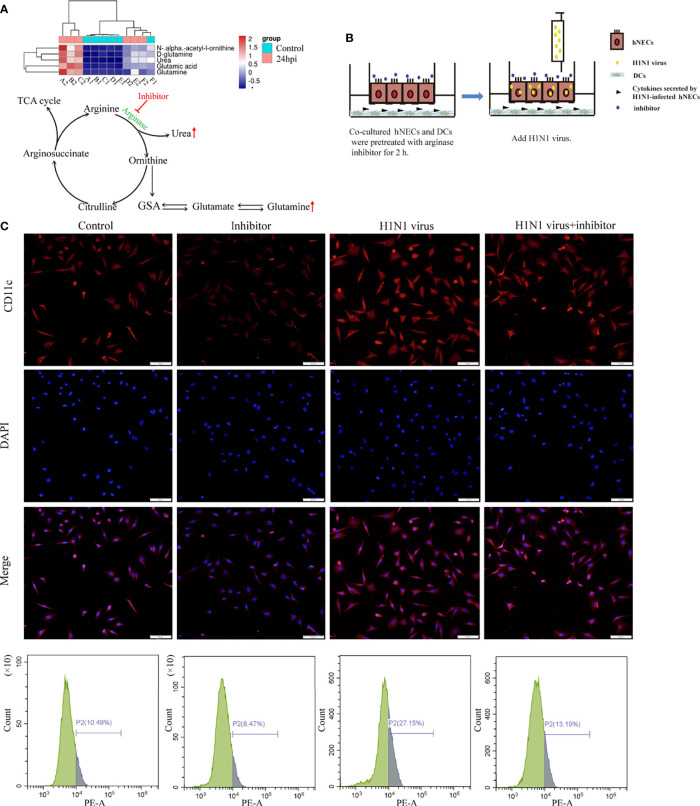
Arginase inhibitor 1 can reduce DCs cell recruitment induced by H1N1 virus-infected hNECs cells. **(A)** Differentially expressed metabolites in the arginine biosynthesis signaling pathway and the pattern map of the signaling pathway. **(B)** Before H1N1 virus infection of hNECs and DCs co-culture system, arginase inhibitor was added for pretreatment for 2h, and then virus infection was carried out. **(C)** H1N1 virus infection in the co-culture system for 24h, the recruitment of DCs was detected by immunofluorescence, the red fluorescence represents CD11c-specific staining, and the blue fluorescence represents nuclear staining (DAPI), bar=50um. The peak graph on the right is the detection of CD11c red fluorescence intensity by flow cytometry.

According to the results of genome sequencing and metabolome sequencing, it can be suggested that the H1N1 virus can cause an abnormal arginine synthesis pathway in the nasal mucosal epithelial cells. Arginine is mainly involved in three processes; urea cycle, nitric oxide synthesis, and polyamine metabolism, and these three processes need to be regulated by a variety of biologically active molecules produced by enzymatic reactions. We analyzed the metabolomic data and found that both urea and glutamine, the downstream metabolites of arginine, were increased, and the production of both urea and glutamine was closely related to the regulation of arginase activity (**Figure 7A**). Therefore, we speculate that arginase plays an irreplaceable role in the regulation of nasal mucosal epithelial metabolic pathways when the nasal mucosal epithelium is attacked by H1N1 virus. Arginase inhibitor 1 is a potent inhibitor of arginase (arginases I and II). We added arginase inhibitor 1 to the co-culture system of nasal mucosal epithelial cells and DCs to test whether arginine metabolism affects the regulation of DCs by nasal mucosal epithelial cells after virus infection (**Figure 7B**). The results showed that arginase inhibitor 1 significantly reduced the recruitment of DCs induced by nasal mucosal epithelial cells after virus infection (**Figure 7C**).

### Arginine Metabolism Is Involved in the Regulation of the Immune Response in Mice Induced by H1N1 Virus Infection

**Figure 8:**
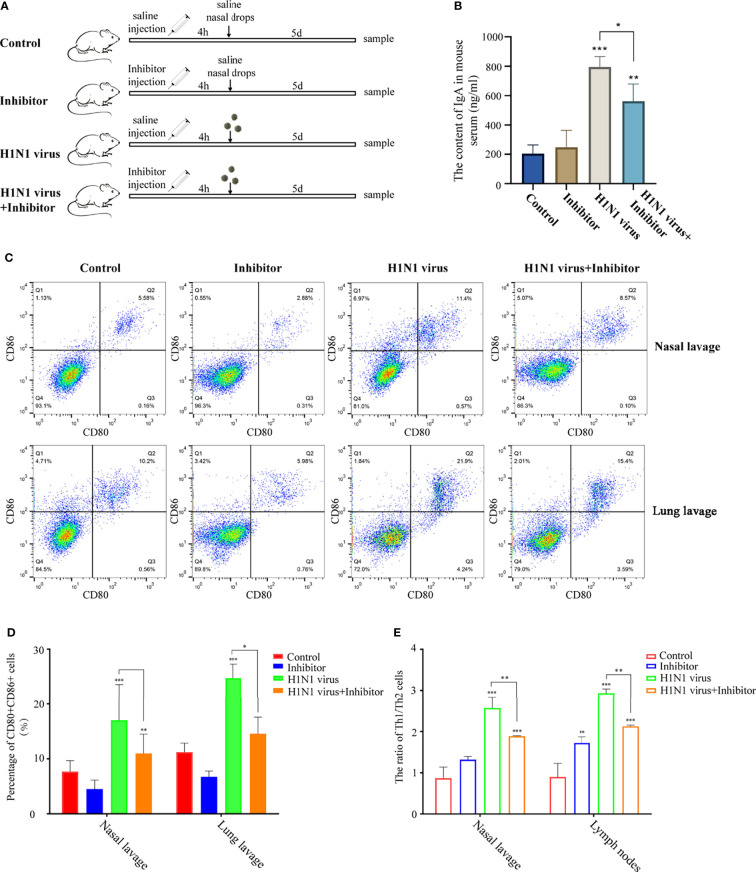
Arginase inhibitor 1 attenuates H1N1 virus infection-induced immune responses in mice. **(A)** Experimental procedure for drug injection in mice. Arrows indicate the drug injection time point.Mice in the control group were injected with the same amount of saline nasal drops at the same time point. **(B)** Elisa was used to detect IgA levels in mouse serum after H1N1 virus infection and arginase inhibitor 1 intervention. **(C)** Flow cytometry was used to detect the proportion of CD80+CD86+ DCs in nasal lavage fluid and lung lavage fluid of mice after H1N1 virus infection and arginase inhibitor 1 intervention. **(D)** The bar graphs show quantification of the flow cytometry results, with each value represents the mean ± SD of three independent experiments. **(E)** Statistical chart of the ratio of Th1/Th2 in nasal lavage fluid and lymphoid tissue of mice detected by flow cytometry. Statistical significance is shown using the Student’s t test analysis, *P < 0.05; **P < 0.01; ***P < 0.001.

To explore whether the regulation of the arginine metabolic pathway can alleviate the immune response in mice induced by H1N1 virus infection, we intravenously injected H1N1 virus-infected mice with arginase inhibitor I (**Figure 8A**). Then, we detected the levels of IgA in the serum of mice after arginine inhibitor I intervention, and the results showed that arginase inhibitor I could alleviate the increased levels of IgA caused by H1N1 virus (**Figure 8B**). At the same time, we detected the proportion of CD80+CD86+DCs in the nasal lavage fluid and bronchoalveolar lavage fluid and the proportion of Th1 and Th2 cells in lymph nodes of mice. The results showed that arginase inhibitor I could significantly reduce the proportions of CD80+CD86+DCs (**Figures 8C, D**) and Th1 cells induced by H1N1 virus infection (**Figure 8E** and **Supplementary Materisal S1**). Considering the above results, we concluded that arginase inhibitor I can interfere with the process of arginine metabolism, and then it can participate in the regulation of the body’s immune response caused by H1N1 virus.

## Discussion

The mucosa lining the upper respiratory tract is an important line of defense against the invasion of pathogens, and it is also an important region of the body that resists influenza virus infection (25). Influenza A virus H1N1 is an influenza virus that causes severe damage to respiratory epithelial cells. It can damage the integrity of respiratory epithelial cells and has strong invasiveness (26, 27). Studies have shown that influenza A virus H1N1 can cause apoptosis of upper respiratory epithelial cells, trigger respiratory mucosal immunity, and cause an inflammatory response in the body (28, 29). However, the specific mechanism by which influenza A virus H1N1 invades the nasal epithelium and elicits an immune response remains unclear.

Nasal mucosal epithelial cells are the first line of defense that the H1N1 influenza virus needs to break through during the process of invasion in the body (30–32). Therefore, when the body is attacked by viruses, the epithelial cells of the nasal mucosa are the most severely damaged. To explore the effect of H1N1 influenza virus on the viability of nasal mucosal epithelial cells, we cultured primary nasal mucosal epithelial progenitor cells *in vitro* and infected them with H1N1 influenza virus. H1N1 influenza virus could significantly impair the activity of nasal mucosal epithelial cells and could normally expand in nasal mucosal epithelial progenitor cells. In addition, consistent with previous research reports, H1N1 influenza virus could significantly promote the apoptosis of nasal mucosal epithelial cells (**Figure 1**). We speculated that the apoptosis of nasal mucosal epithelial cells is one of the body’s self-protection strategies. A large number of cytokines are also released during the apoptosis of nasal mucosal epithelial cells, which will promote the clearance of viruses.

The respiratory mucosal immune system is an important part of the mucosal immune system (33). When the influenza virus invades, it will open the first important line of defense involved in protection of the body. When the influenza virus breaks through the mucosal barrier, it adsorbs and invades the respiratory epithelial cells, resulting in a large number of cytokines, such as interferons, pro-inflammatory factors, and chemokines (33). Some experimental studies have shown that respiratory epithelial cells can significantly increase the expression of inflammatory factors (34). Therefore, we infected the nasal mucosal epithelial cells differentiated *in vitro* with H1N1 influenza virus, and observed the changes in cytokines, chemokines, interferons, and other inflammation-related factors in the nasal mucosal epithelial cells. In addition to interferon alpha and gamma, there are a large number of interleukins, and the expression of chemokines is increased. This indicates that H1N1 virus may trigger the inflammatory storm of nasal mucosal epithelial cells to resist virus invasion (**Figure 2**).

Studies have shown that when the nasal mucosal epithelial cells are attacked by bacteria, viruses, or allergens, the nasal mucosal epithelial cells will release a large number of cytokines, especially chemokines, to recruit more immune cells to the lower part of the mucosa to clear the pathogens (35). Mature DCs can respond to the invasion of influenza virus, carry out the capture and presentation of viral antigens, and activate the activation of the mucosal immune system (36). Our study showed that co-culture of H1N1 virus-infected nasal mucosal epithelial cells with DCs significantly enhanced the recruitment and maturation of DCs (**Figure 3**). Therefore, we conclude that stimulation of DC recruitment and activity is also one of the strategies of nasal mucosal epithelial cells against H1N1 influenza virus.

To explore the mechanism by which H1N1 virus-infected nasal mucosal epithelial cells induce downstream immune responses, we performed genome and metabolome sequencing. The signal pathway enrichment analysis data showed that the IL-17 signaling pathway changed most obviously in the nasal mucosa epithelial cells after virus infection (**Figures 4**, **5**), which was consistent with the results of our previous study of inflammatory factors (**Figure 2**). Studies have shown that IL-17 can mediate protective immunity against Bordetella pertussis nasal infection by mobilizing neutrophils, especially Siglec-F neutrophils (37). Although some researchers claim that IL-17 can promote the recruitment of submucosal immune cells (38), there is no direct evidence that IL-17 plays a role in the recruitment of DCs. In the virus-stimulated nasal mucosa, in addition to IL-17, TNF-α and TGF-β signaling pathways also cause significant changes. The regulatory role of these signaling pathways on the functions of DCs and other immune cells needs to be further verified, which is also one of the limitations of this study.

Combined with the analysis results of genome sequencing and metabolome sequencing, we screened out the two overlapping arginine synthesis signaling pathways for subsequent verification. Arginine metabolism is an important regulator of various innate and acquired immune responses (39). Studies have shown that the main enzymes involved in the metabolic synthesis of arginine are arginase and nitric oxide (NO) synthase (40, 41). However, our metabolomic analysis data showed that urea and glutamine, both downstream metabolites of arginine regulated by arginase, were significantly up-regulated in nasal epithelial cells after virus stimulation (**Figure 7A**). Therefore, we used arginase inhibitors to inhibit arginase activity, and then we verified the regulation of arginine metabolism on the function of downstream DCs in nasal mucosal epithelial cells. The results of *in vitro* and *in vivo* experiments showed that the intervention of arginine metabolism could reduce the recruitment and maturation of DCs in nasal mucosal epithelial cells infected with H1N1 virus, decrease the production of IgA, and reduce the ratio of Th1/Th2 cells.

In this study, we mainly explored the changes in cytokine secretion, metabolites, and signaling pathways in nasal mucosal epithelial cells induced by H1N1 virus infection, and we elaborated the role of arginine metabolism in H1N1 virus-induced nasal mucosal immunity. Our study suggests that arginine metabolism-related immune regulatory nodes may be therapeutic targets for combating influenza virus.

## Conclusion

H1N1 influenza virus can induce apoptosis and cytokine storm in nasal mucosal epithelial cells. Furthermore, H1N1 influenza virus promotes the recruitment and maturation of submucosal DCs by causing abnormal arginine metabolism in nasal mucosal epithelial cells.

## Data Availability Statement

The original contributions presented in the study are publicly available. This data can be found here: https://www.ncbi.nlm.nih.gov/Traces/study/?acc=PRJNA813174.

## Ethics Statement

The studies involving human participants were reviewed and approved by the Ethics Committee of Shandong Provincial Hospital Affiliated to Shandong First Medical University. The patients/participants provided their written informed consent to participate in this study. The animal study was reviewed and approved by the Ethics Committee of Shandong Provincial Hospital Affiliated to Shandong First Medical University.

## Author Contributions

FZ, drafted the important content of the manuscript and explained it, and carried out rigorous conception and design of the subject. ZT and XZ, carried out a detailed analysis of the data in the article. RX, XB, and MW, carried out the collection of clinical samples. LS and NG, conducted experimental operations. In addition, MX and CL, provided the subject ideas and careful proofreading of the manuscript. All authors contributed to the article and approved the submitted version.

## Funding

This work was supported by the grants from the Medical Science and Technology Innovation Center, Shandong First Medical University and Shandong Academy of Medical Sciences, the National Natural Science Foundation of China (#81770979, #81900922), Natural Science Foundation of Shandong Province (#ZR2019BH019), Joint Fund of Shandong Province (#ZR202108050034) and Taishan Scholar Foundation of Shandong Province (#tsqn201812134).

## Conflict of Interest

The authors declare that the research was conducted in the absence of any commercial or financial relationships that could be construed as a potential conflict of interest.

## Publisher’s Note

All claims expressed in this article are solely those of the authors and do not necessarily represent those of their affiliated organizations, or those of the publisher, the editors and the reviewers. Any product that may be evaluated in this article, or claim that may be made by its manufacturer, is not guaranteed or endorsed by the publisher.

## Supplementary Material

Supplementary Figure 1 Arginase inhibitor 1 decreased the proportion of TH1 cells in mice caused by H1N1 viral infection. Flow cytometry was used to detect the proportion of IFN-γ+IL-4+ in nasal lavage fluid and lung lavage fluid of mice after H1N1 virus infection and arginase inhibitor 1 intervention.
